# Novel birch pollen specific immunotherapy formulation based on contiguous overlapping peptides

**DOI:** 10.1186/2045-7022-3-17

**Published:** 2013-06-01

**Authors:** Céline Pellaton, Yannick Perrin, Caroline Boudousquié, Nathalie Barbier, Jacqueline Wassenberg, Giampietro Corradin, Anne-Christine Thierry, Régine Audran, Christophe Reymond, François Spertini

**Affiliations:** 1 Division of Immunology and Allergy, Centre Hospitalier Universitaire Vaudois (CHUV), Rue du Bugnon, Lausanne, 1011, Switzerland; 2 Anergis SA, Lausanne, Switzerland; 3 Department of Biochemistry, University of Lausanne, Lausanne, Switzerland

**Keywords:** IgE, Peptides, Immunotherapy, Birch, Pollen

## Abstract

**Background:**

Synthetic contiguous overlapping peptides (COPs) may represent an alternative to allergen extracts or recombinant allergens for allergen specific immunotherapy. In combination, COPs encompass the entire allergen sequence, providing all potential T cell epitopes, while preventing IgE conformational epitopes of the native allergen.

**Methods:**

Individual COPs were derived from the sequence of Bet v 1, the major allergen of birch pollen, and its known crystal structure, and designed to avoid IgE binding. Three sets of COPs were tested *in vitro* in competition ELISA and basophil degranulation assays. Their *in vivo* reactivity was determined by intraperitoneal challenge in rBet v 1 sensitized mice as well as by skin prick tests in volunteers with allergic rhinoconjunctivitis to birch pollen.

**Results:**

The combination, named AllerT, of three COPs selected for undetectable IgE binding in competition assays and for the absence of basophil activation *in vitro* was unable to induce anaphylaxis in sensitized mice in contrast to rBet v 1. In addition no positive reactivity to AllerT was observed in skin prick tests in human volunteers allergic to birch pollen. In contrast, a second set of COPs, AllerT4-T5 displayed some residual IgE binding in competition ELISA and a weak subliminal reactivity to skin prick testing.

**Conclusions:**

The hypoallergenicity of contiguous overlapping peptides was confirmed by low, if any, IgE binding activity *in vitro*, by the absence of basophil activation and the absence of *in vivo* induction of allergic reactions in mouse and human.

**Trial registration:**

ClinicalTrials.gov NCT01719133

## Introduction

Allergen specific immunotherapy (SIT) is the only etiologic treatment available for IgE-mediated allergy [1]. SIT consists in the administration of incremental doses of allergen extracts progressively leading to immune and clinical tolerance to the allergen. The efficacy of SIT has been largely demonstrated [2-4]. However, a number of side effects are observed during conventional SIT [5] as well as during ultrarush immunotherapy, where up to 30% of patients develop anaphylactic reactions, sometimes severe or even fatal [6]. While the duration of treatment may potentially be largely shortened, the current duration lasting three to five years of monthly injections depending on the allergen sensitivities. Different innovative strategies recently aimed to improve both efficacy and safety of SIT. A first approach consisted in improving existing formulations by using, as adjuvants, TLR agonists including monophosphoryl lipid A (MPL) [7-9], DNA immunostimulatory sequences (CpG) [10] or viral particles combined to CpG [11] in order to mainly stimulate a TH1 response to a limited amount of whole allergen extracts. In another strategy targeting specifically the allergen formulation, a combination of recombinant major allergens appeared to improve rhinitis combined score in patients allergic to grass pollen [12]. Interestingly immunotherapy with the recombinant major birch pollen allergen Bet v 1 proved as efficient as purified native Bet v 1 or birch pollen extract [12,13], showing that a single allergen may potentially substitute for the whole extract. A third line of development explored the use of products with hypoallergenic potential as demonstrated by reduced IgE binding capacity but conserved T cell recognition, and involved mainly T cell epitope peptides and allergen fragments of various lengths [14-18] or point mutations on IgE epitopes [19].

We previously performed a phase I trial based on the subcutaneous injection of synthetic contiguous overlapping peptides (COPs) from the major bee venom allergen phospholipase A2 (PLA2) [20]. Clinical tolerance was excellent. Immunogenicity was marked by enhancement of PLA2 specific IgG4 levels, T cell hyporesponsiveness, TH1 cytokine deviation and PLA2 peptide specific IL-10 production. Based on this approach, we describe in this paper the design and first clinical application of novel sets of COPs derived from the birch major allergen Bet v 1. Among allergic patients hypersensitive to tree pollens, skin prick tests to birch pollen extract are positive in a large majority of patients and most of them have developed specific IgE to Bet v 1, a member of the PRP-10 family of plant defense proteins. In addition, hypersensitivity to birch pollen is very often linked to allergies to other trees of the *Fagales* order and at the origin of crossreactive hypersensitivities to a large array of food allergens belonging to *Rosaceae, Juglandaceae, Corylaceae, Apiaceae* or *Solanaceae* families [21-24].

Our aim in this study was to evaluate COPs hypoallergenicity *in vitro* (based on their IgE binding capacity) and *in vivo* (based on their capacity to induce an allergic reaction in animals or a positive skin test in Bet v 1 allergic volunteers) as a preliminary study to a therapeutic phase I clinical trial in patients with allergic rhinitis to birch pollen.

## Material and methods

### Peptides synthesis and purification

Three sets of COPs composed of peptides T1-T2-T3, T4-T5 and T6-T7-T8 respectively (Table 1), all mapping the whole sequence of Bet v 1 were synthesized according to GLP recommendations by solid phase fmoc chemistry on an Applied Biosystems 431A Peptide Synthesizer (Perkin Elmer, Foster City, Calif) and purified as described [25]. Analytic HPLC and mass spectrometry were used to assess the purity of each peptide (>90%). Peptides were resuspended in water (2 mg/ml) and frozen at -20°C in aliquots. The equimolar combination of T1, T2 and T3, composing the finally selected product, was named AllerT.

**Table 1:** Sequences and physico-chemical characteristics of Bet v 1-derived synthetic contiguous overlapping peptides (aa sequence refers to Bet v 1.01-A, SwissProt P15494.2)

**COP name**	**Sequence**	**pI**	**Mw**
**T1**: aa 2-50	GVFNYETETTSVIPAARLFKAFILDGDNLFPKVAPQAISSVENIEGNGG	4.36	5295.93
**T2**: aa 48-118	NGGPGTIKKISFPEGFPFKYVKDRVDEVDHTNFKYNYSVIEGGPIGDTLEKISNEIKIVATPDGGSILKIS	5.72	7742.76
**T3**: aa 106-160	VATPDGGSILKISNKYHTKGDHEVKAEQVKASKEMGETLLRAVESYLLAHSDAYN	6.29	6001.72
**T4**: aa 2-85	GVFNYETETTSVIPAARLFKAFILDGDNLFPKVAPQAISSVENIEGNGGPGTIKKISFPEGFPFKYVKDRVDEVDHTNFKYNYS	5.24	9348.49
**T5**: aa 65-160	FKYVKDRVDEVDHTNFKYNYSVIEGGPIGDTLEKISNEIKIVATPDGGSIL	5.77	6001.72
	KISNKYHTKGDHEVKAEQVKASKEMGETLLRAVESYLLAHSDAYN		
**T6**: aa 2-49	GVFNYETETTSVIPAARLFKAFILDGDNLFPKVAPQAISSVENIEGNG	4.36	5141.77
**T7**: aa 44-118	NIEGNGGPGTIKKISFPEGFPFKYVKDRVDEVDHTNFKYNYSVIEGGPIGDTLEKISNEIKIVATPDGGSILKIS	5.24	8156.19
**T8**: aa 103-160	IKIVATPDGGSILKISNKYHTKGDHEVKAEQVKASKEMGETLLRAVESYLLAHSDAYN	7.03	6356.22

aa : amino acids.

### Animals

Four weeks-old female BALB/c mice (H-2^d^) were obtained from Harlan (AD Horst, The Netherlands) and used at the age of 6–8 weeks.

### Sensitization of mice and challenge

Mice were sensitized with subcutaneous injections of 0.1 μg rBet v 1 adsorbed on 1 mg Aluminum Hydroxide (Sigma Chemicals, St-Louis, MO, USA) up to six times at 2 weeks intervals as previously described [26]. Two weeks later (D84), mice were challenged i.p. either with 30 μg rBet v 1 (BIOMAY, Vienna, Austria) or 190 μg AllerT. Rectal temperature was recorded before, 15, 30, 45 and 60 min after challenge with a digital thermometer (Terumo, Tokyo, Japan). Sera were collected the day before each treatment.

### Mouse isotypic anti-rBet v 1 IgE and IgG response

Mouse serum IgE, IgG1 and IgG2a antibody responses were determined by ELISA as previously described [26,27]. Briefly, 96-well Nunc Maxisorp® immunoplates (Life Technologies, Basel, Switzerland) were coated with 5 μg/ml rBet v 1. After blocking with 1% BSA, plates were incubated with optimal dilutions of mouse sera, namely 1:5 for IgE, 1:200 for IgG1, 1:50 for IgG2a. Biotinylated rat anti-mouse IgE (2 μg/ml), IgG1 or IgG2a (167 ng/ml) (PharMingen, BD-Biosciences, San Diego, CA) were used as secondary antibodies, revealed with extravidin alkaline phosphatase and 4-NPP substrate (Sigma Diagnostic Inc., St-Louis, MO, USA) and OD read at 405 nm using a microplate reader (Dynatech laboratories, Chantilly, VA, USA). A titration of purified mouse IgE (27–74, PharMingen) on microwells coated with 2 μg/ml rat anti-mouse IgE (R35-72, PharMingen) was used to convert OD in IgE concentration.

### Degranulation assays on rat basophil lines

Degranulation assay was performed as previously described [28]. RBL-2H3 cells were plated in 96-well tissue culture plates (4×10^4^ cells/well) overnight. Passive sensitization of RBL-2H3 cells was carried out with sera from rBet v 1-sensitized mice at a final dilution of 1:30 for 2 h. Unbound antibodies were removed by washing the cell layer twice in Hanks’ balanced salt solution (Sigma-Aldrich, St-Louis, MO, USA). Degranulation of RBL cells was induced by adding rBet v 1, individual COP T1, T2, T3 or AllerT diluted in HBH buffer (HBSS with 1% BSA and 10 mM HEPES) for 1h at 37°C at indicated concentrations. β-hexosaminidase activity was analyzed by incubating 50 μl of cell supernatant with 50 μl of 1.3 mg/ml 4-Nitrophenyl N-acetyl-β-D-glucosaminide (Sigma) in citrate buffer (0.1M, pH 4.5) for 1 h at 37°C. The reaction was stopped by addition of 100 μl glycine buffer (0.2M glycine, 0.2M NaCl, pH 10.7) and optical densities (OD) measured at 405 nm.

### Competition ELISA for rBet v1 specific hu IgE

Dilutions (1:5 to 1:10) of sera from 6 birch pollen allergic patients (specific Bet v 1 IgE >15.9 kU/L, Phadia, Uppsala, Sweden) were incubated 1h with tenfold serial dilutions of either individual COPs (T1, T2, T3; T4, T5), COPs combinations AllerT, T4-T5, T6-T7-T8 or rBet v 1, starting at 10^-5^ M, then distributed to rBet v 1 coated and BSA 1%-blocked 96-well immunoplates (Nunc Maxisorp®Life Technologies, Basel, Switzerland) for 2 h RT or overnight at 4°C. Successively and with washings between steps, plates were incubated 1 h at RT with biotinylated mouse anti-huIgE (Pharmingen, 5 μg/ml, 1 h at RT), Streptavidin-HRP or -alkaline phosphatase (BD, 1:1000 or 1:8000, respectively, 30 min at RT) and TMB (BD OptEIA, ) or 4-NPP. OD were read at 450 nm, 630 nm and 405 nm, respectively. Inhibition of IgE binding to Betv 1 was calculated as: (OD with competitor minus ODmin)/(OD without competitor minus ODmin) × 100, where ODmin was determined in wells without serum.

### Human basophil activation assay

The direct assay was performed using the Basotest™ assay according to manufacturer’s instructions (Orpegen Pharma, Heidelberg, Germany). Heparinized blood (100 μl) was first incubated with stimulation buffer for 10 min at 37°C, and then with 100 μl allergen solution diluted in a saline solution for 20 min at 37°C. A dose–response curve was performed with ten-fold dilutions of rBet v 1 or AllerT. Formyl methionyl leucyl phenylalanine was used as a positive control and PBS as a negative control. The activation process was stopped by incubating blood samples at 4°C for 10 min. Samples were stained for 20 min at 4°C with 20 μl anti-IgE-PE mAb and anti-CD63-FITC. Erythrocytes were subsequently removed by adding 2 ml lysis solution (Becton-Dickinson) and two washes with PBS. Cells were resuspended in 200 μl PBS and at least 10^3^ basophils for each sample were acquired within 1 h on a FASCalibur, Becton-Dickinson. Results were expressed as the percentage of basophils (IgE^+^ cells) expressing CD63. A cut-off ≥15% CD63^+^ IgE^+^ basophils was chosen as the lower limit of positivity according to the manufacturer. In indirect assay, 100 μl of heparinized blood from a non-allergic subject was pre- incubated 2 h at 37°C with 25 μl of plasma or sera from allergic subjects before performing the Basotest™ assay as described above.

### Degranulation assays on humanised basophil cell line RBL703/21

The assay was adapted from Ladics et al. [29] and Blanc et al. [30]. RBL cells transfected with human FcϵRI (RBL703/21, kind gift of Dr L. Vogel, Paul-Ehrlich Institute, Germany) were plated at 10^5^ cells/well in medium (MEM containing glutamine and penicillin/streptomycin) and incubated overnight with sera from subjects with class 4, 5 and 6 IgE levels to birch pollen extract as determined by ImmunoCAP (Phadia, Uppsala, Sweden). Human serum was diluted 1/2 with 4M glucose and incubated for 45 min at 56°C, centrifuged and diluted 1/5 with MEM medium before addition to RBL703/21 cells. After washing with Tyrode’s buffer (137 mM NaCl, 2.7 mM KCl, 0.4 mM Na phosphate, 0.5 mM MgCl_2_, 1.4 mM CaCl_2_, 10 mM Hepes, 5.6 mM glucose, 0.1% BSA), cells were incubated for one hour at 37°C with various concentrations of rBet v 1, COPs or 0.5 μg/ml goat anti-human IgE heavy chain antiserum (Nordic Immunology Laboratories, Eidhoven, NL) in Tyrode’s buffer containing 50% deuterium oxide or Tyrode’s buffer alone as control. Supernatants (30 μl) were incubated in duplicates with 50 μl of substrate solution (1.3 mg/ml p-nitrophenyl-b-D-2-acetamido-2-deoxyglucopyranozide from Sigma in 0.1 M citrate pH 4.5) for 1 hour at 37°C. Reaction was stopped by the addition of 100 μl of 0.2 M glycin (pH 10.7). OD was measured at 405 nm (reference filter at 620 nm). β-hexosaminidase release was calculated using the following formula: (OD _sample_-OD_buffer_) / (OD_anti-IgE_-OD_buffer_) × 100.

### AN002 phase I clinical trial: study population

The AN002 phase I clinical trial was a single center, open and controlled study conducted at the Centre Hospitalier Universitaire Vaudois (CHUV) in Lausanne. Volunteers (18–45 years) with at least one season history of birch pollen rhinoconjunctivitis or asthma were recruited outside of the birch pollen season. Volunteers treated with antihistamines or medication with antihistamine activity less than 15 days prior to skin prick testing were excluded as well as volunteers with peak expiratory flow (PEF) <20% of volunteer’s best PEF value. Birch pollen sensitization was documented 1) by positive skin prick test to birch pollen extract (Aquagen SQ, ALK Abello) and to rBet v 1 (BIOMAY, Vienna, Austria), and 2) positive birch pollen and rBet v 1-specific serum IgE (>0.35 kU/L by ImmunoCAP assay). Inferior limits of specific IgE to determine allergy classes 1 to 6 are 0.35, 0.7, 3.5, 17.5, 50 and 100 kU/L respectively. The clinical research protocol was approved by the Ethical Review Board of the Faculty of Biology and Medicine, Lausanne, and by the Swiss National Regulatory Authority (Swissmedic) and posted on the ClinicalTrials.gov site under the study number NCT01719133. The primary objective of the study was to determine the reactogenicity of selected COPs by skin prick tests.

### Skin prick testing

Individual T1 to T5 COPs, AllerT and T4-T5 mix were evaluated *in vivo* by skin prick tests following EAACI guidelines [31]. For individual COP or mixes, a 20 μl drop was applied onto the forearm of volunteers at 3 different dilutions in sterile PBS (10 μM, 1 μM, 0.1 μM) and compared to skin prick tests with serial ten-folds dilutions (in NaCl 0.9%) of birch pollen extract (Aquagen SQ, ALK-Abello, Horsholm, Denmark, 100’000 SQ, 10’000 SQ and 1000 SQ) and rBet v 1 (1 μM, 0.1 μM, and 0.01 μM).

### Statistical analysis

In animal studies, results are expressed as mean ± SD of 6–10 mice/group. Each experiment was done at least three times. Significance of differences between means was determined using Student’s or non-parametric *t* test. * = p<0.05; **= p<0.01; *** = p<0.001. Analysis was done using the Prism 4.00 software for Windows (GraphPad Software Inc., San Diego, CA, USA). Data analysis of clinical trial AN002 was descriptive.

## Results

### Design of the Contiguous Overlapping Peptides (COPs)

Bet v 1 is a 159 amino acid (aa) long protein present as a mix of isoforms in pollen extracts and differing by a few amino acids [32]. Based on the sequence X15877 of rBet v 1.01A, the most represented Bet v 1 isoform [15], we devised three sets of COPs with potentially disrupted IgE conformational epitopes. We based our strategy on Bet v 1 sequence comparison between *Fagales* species showing regions of particularly high sequence conservation that may possibly contain IgE epitopes as proposed by Spangfort et al. [33]. Three conformational regions are at high risk of binding IgE. A first region includes aa 97–121 combined to aa 131–141; a second region encompasses aa 15–31 and a third region involves a loop located at aa 41–52 homologous to the GTP-binding sequence GXGXXG [34]. On the other hand, T cell epitopes are scattered throughout the Bet v 1 sequence except for amino acids 48–59, containing the potential GTP binding site GXGXXG [35]. The sets of COPs T1-T2-T3 and T4-T5 encompass the complete Bet v 1 sequence (Table 1). In order to conserve all potential T cell epitopes, COPs were overlapped at their extremities by 3, 13 and 21 aa for T1/T2, T2/T3 and T4/T5 respectively. A third set of COPs, namely T6-T7-T8 was derived from T1, T2 and T3 with modified extremities (Table 1). In particular, the C-terminus of T6 was one amino acid shorter than T1. T7 has four amino acids added at the N-terminus compared to T2, and T8 has three amino acids added also at the C-terminus. COPs were either used separately or mixed in equimolar amounts in further experiments.

### Analysis of human Bet v 1 specific IgE binding to COPs by competition assay

**Figure 1:**
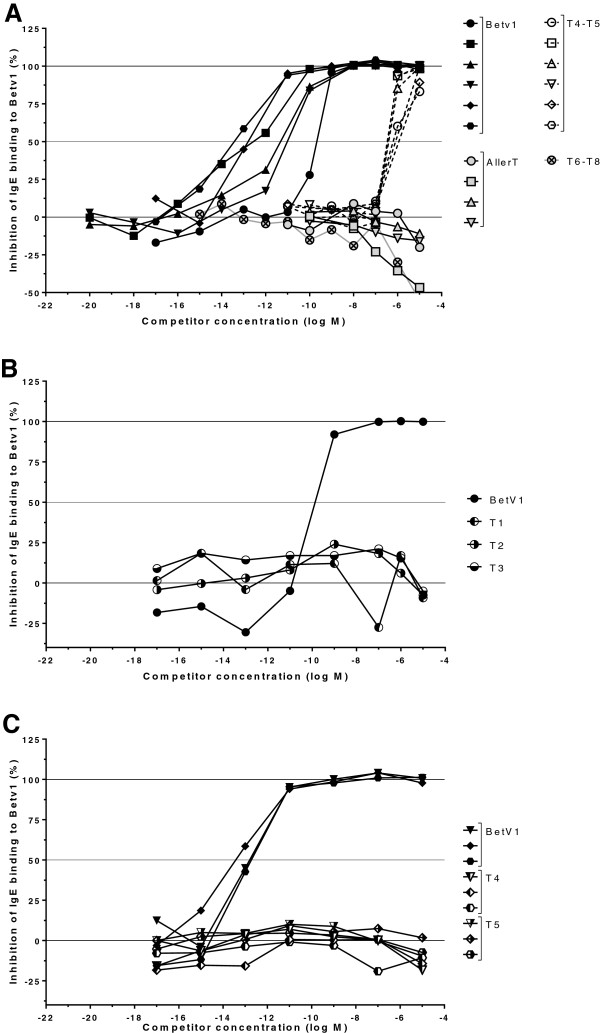
**Human IgE competition assays.** rBet v 1-specific IgE were measured by ELISA in sera from individual birch pollen allergic patients pre-incubated with increasing concentrations of either rBet v 1 or COPs as indicated on the X axis. Panel **A**: Sera from six allergic patients (indicated by various symbol shapes) were pre-incubated with either rBet v 1 (black symbols), AllerT (grey symbols), T4-T5 (open symbols) and T6-T8 (crossed circles). Panel **B**: Individual COPs T1, T2 and T3 were tested separately with serum from a representative patient as indicated in the Figure (control rBet v 1, black circles). Panel **C**: Sera from three allergic individuals was reacted with either T4 or T5 (black & white symbols) alone or control rBet v 1 (black symbols). Inhibition of binding is expressed as the inverse of the percentage of residual IgE binding to Bet v 1.

COPs were first tested *in vitro* for birch specific IgE binding by competition ELISA. Soluble rBet v 1 inhibited serum IgE binding with EC_50_ ranging from 10^-14^ to 10^-10^ M, (Figure 1A). COPs T1, T2 and T3 either in equimolar combination (AllerT) (Figure 1A) or individually (Figure 1B) showed no competitive activity up to 10^-5^ M. The same result was obtained using COPs T6, T7 and T8 in combination (Figure 1A), indicating that the precise ending of the COPs was not essential for reducing IgE binding. Results were comparable with T4 and T5 individually as competitors (Figure 1C). Surprisingly, the combination of T4-T5 as an equimolar mix did inhibit IgE binding with EC_50_ in the range of 10^-7^-10^-8^ M, i.e. at least at concentration 10^4^-fold higher than rBet v 1 (Figure 1A). The fact that competition reaches completion suggests partial refolding due to interaction of T4 and T5 in solution, thus re-creating the original IgE epitope(s).

### Effect of AllerT on mouse IgE-induced basophil degranulation

**Figure 2:**
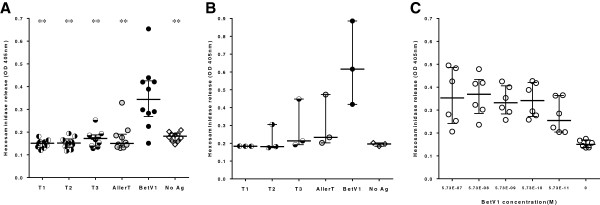
**Degranulation of rat basophil lines induced by AllerT and its respective individual COPs T1, T2 and T3.** Beta-hexosaminidase release was measured from RBL-2H3 cells loaded with sera obtained from r Bet v 1-sensitized mice and stimulated in panel **A.** with 5.7 nM rBet v 1, T1, T2, T3, AllerT or left unstimulated, n=10; in panel **B**: with 57 nM rBet v 1, T1, T2, T3, AllerT or left unstimulated, n=3; panel **C**: Beta-hexosaminidase release from RBL-2H3 cells loaded with 2 sera (dilutions 1:10, 1:50 and 1:100) obtained from r Bet v 1-sensitized mice and stimulated with serial concentrations of Bet v 1. Degranulation was measured using a colorimetric assay and is expressed in OD. Median and quartiles are indicated as horizontal bars. Data were analyzed by a non-parametric paired t test and compared to rBetv1. **p <0.01.

The capacity of COPs to induce basophil degranulation was characterized using rat basophil leukemia cells (RBL) [28]. Upon stimulation with rBet v 1, hexosaminidase accumulated significantly in the supernatant from RBL cells primed with sera from Bet v 1-sensitized mice (see below). On the contrary, AllerT as well as individual COPs were unable to induce hexosaminidase release even at ten-fold (5.7 nM, panel A) and hundred-fold (57 nM, panel B) higher concentrations than optimal rBet v 1 (0.57 nM, panel C) (Figure 2).

### Effect of AllerT challenge on Bet v 1 sensitized mice

**Figure 3:**
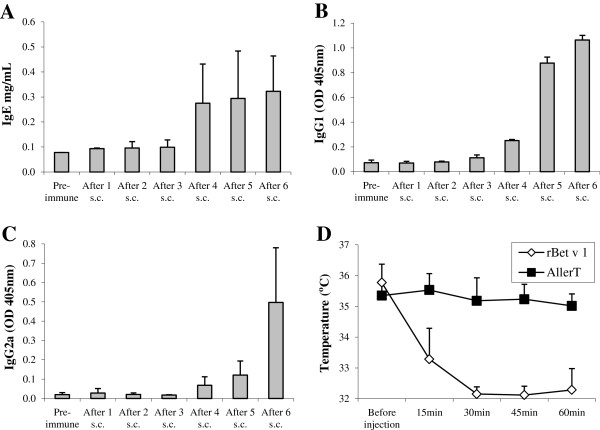
**BALB/c mice sensitization and in vivo challenge.** Mice were sensitized to rBet v 1 by injecting 0.1 μg rBet v 1 adsorbed to 1 mg Aluminum hydroxide. rBet v 1-specific IgE (panel **A**), IgG1 (panel **B**) and IgG2a (panel **C**) were measured in mice serum harvested immediately before the next injection. Results were expressed as means ± SD. Panel **D**. Rectal temperature was recorded at indicated time points following 30 μg rBet v 1(◊) or 190 μg AllerT (■) i.p. challenge at day 84 of the immunization protocol.

Mice were sensitized by repeated injections of rBet v 1 (0.1 μg in Aluminum Hydroxide) as described previously [28]. Bet v 1 specific IgE increased markedly after the fourth injection to level thereafter (Figure 3A). IgG1 and IgG2a levels started to increase after the fourth injection and steadily increased up to the last injection (Figure 3B and C). Clinical hypersensitivity of mice was tested by injecting i.p. 30 μg rBet v 1, using rectal temperature as a surrogate marker of anaphylaxis [26]. As seen in Figure 3D, a marked temperature drop was observed within 30 minutes after i.p. injection of rBet v 1. In contrast, injection of up to 190 μg of AllerT did not lead to any temperature shift, strongly indicating the absence of clinically relevant IgE binding to AllerT.

### Absence of detectable human basophil activation by COPs

**Figure 4:**
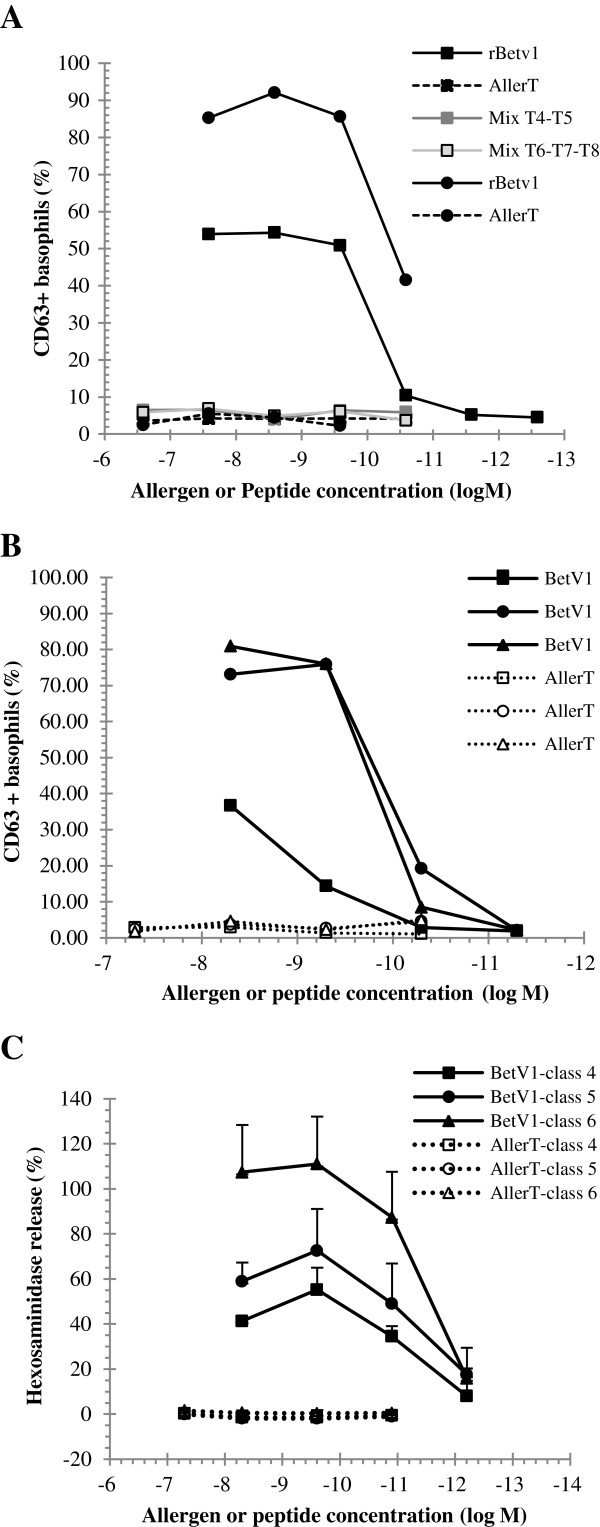
**Human basophil degranulation assay.** Panel **A**: Basophils from two subjects allergic to birch (circle and square symbols) were activated by rBet v 1, AllerT, T4-T5 or T6-T7-T8 mixes at indicated concentrations. Panel **B**: Basophils from a non-allergic donor were pre-incubated with sera from 3 subjects allergic to birch (circles, squares and triangles) and incubated with the indicated concentrations of rBet v 1 and AllerT. Degranulation is expressed as the percentage of CD63 positive basophils. Panel **C**: Rat basophils expressing hu FcϵRI were pre-incubated with sera from 10 subjects allergic birch and reacted with either rBet v 1 or AllerT (filled and open symbols respectively). Results were grouped by IgE class levels as determined by the ImmunoCAP assay (classes 4 to 6; n= 3 or 4 sera per class). Hexosaminidase release is expressed as the percentage of release induced by anti-total IgE antiserum.

In preparation for application to human subjects, AllerT COPs and rBet v 1 were compared for their capacity to induce human basophil degranulation. In a direct Basotest™ assay, blood was collected from patients allergic to birch pollen and degranulation due to IgE crosslinking was tested in response to COPs mixes, namely T1-T2-T3, T4-T5 or T6-T7-T8 (Figure 4A). None of the COP mixes was able to induce functional IgE crosslinking at any concentration tested (up to 1 μM), whereas rBet v 1 induced 50% degranulation already at about 0.1 nM. Thus AllerT displays an at least 10^4^-fold lower capacity to activate basophils from allergic patients.

In an alternative assay, basophils from a non-allergic donor were pre-incubated with sera from birch pollen allergic patients before being incubated with rBet v 1 in an indirect Basotest™ assay (Figure 4B). Degranulation of basophils loaded with IgE occurred at different Bet v 1 concentration, with EC_50_ ranging from about 0.01 to over 1 nM. Again no basophil degranulation was detected upon AllerT addition up to about 100 nM.

Finally, AllerT was tested against IgE from ten patients allergic to birch pollen in an hexosaminidase release assay using huFcϵRI transfected RBL [29]. Degranulation results were grouped by IgE serum levels (ImmunoCAP classes, Figure 4C). Degranulation by rBet v 1 was clearly IgE dose-dependent. A maximal response was reached with a dose of 10^-9^ M rBet v 1, while no degranulation was induced by AllerT up to 10^-7^ M in combination with any of the 10 sera. Taken together these results confirmed that the COPs were unable to crosslink IgE.

### Skin prick testing of birch pollen sensitive volunteers (AN002 clinical trial)

Twenty subjects, 7 males and 13 females, mean age 30.1 years (range 23–45 years) (Table 2), were selected for previous history of birch pollen allergy as well as positive skin prick tests to birch pollen extract (Aquagen SQ, ALK Abello). Skin prick tests with either birch pollen extract (100’000 SQ) or rBet v 1 (1 μM) were positive in all 20 volunteers (data not shown), namely resulted in >3 mm wheal diameter with erythema [31]. Lowering allergen concentration by 10 fold resulted in positive reactions in 85% and 80% of patients for birch pollen extract and rBet v 1 respectively (Table 3), whereas a further 10-fold dilution almost abolished skin prick test reactivity. T1, T2, T3 COPs either alone or in combination were unable to elicit skin prick test positive reactions even at a 10 μM concentration. These data thus indicate that AllerT and individual COPs T1 to T3 were at least 100-fold less reactive *in vivo* than rBet v 1.

**Table 2:** AN002 clinical trial: volunteers’ characteristics

**Parameters**	**Characteristics**
Number of volunteers	20
Mean age (range)	30.1 years (23–45)
Male /Female (nb/nb)	7 / 13
Allergic asthma (nb)	7
Allergic rhinitis (nb)	20
Mean peak expiratory flow rate (% predicted value, L/min) (range)*	93.75 (61–117)
Mean anti-birch pollen IgE (kU/L) (range)	29.34 (0.7 – 100)
Mean anti-rBet v 1 IgE (kU/L) (range)	29.48 (0.35 - 100)

*Mean of three successive measurements.

**Table 3:** Skin prick test results according to EAACI guidelines

**A**			
**Positive skin reactions (≥3 mm wheal and flare present)**			
	**Dilutions**		
	**1:1**	**1:10**	**1:100**
Birch (100’000 SQ)	20	17	1
r Bet v 1 (1 μM)	20	16	2
T1 (10 μM)	0	0	0
T2 (10 μM)	0	0	0
T3 (10 μM)	0	0	0
AllerT (10 μM)	0	0	0
T4 (10 μM)	0	0	0
T5 (10 μM)	0	0	0
Mix T4-T5 (10 μM)	0	0	0
**B**			
**0 mm <Skin reaction < 3 mm**			
	**Dilutions**		
	**1:1**	**1:10**	**1:100**
T1 (10 μM)	2	0	0
T2 (10 μM)	0	0	0
T3 (10 μM)	0	1	0
AllerT (10 μM)	0	0	0
T4 (10 μM)	0	0	1
T5 (10 μM)	1	0	0
Mix T4-T5 (10 ^2^ μM)	6	0	1

The same result was obtained with the combination of T4 and T5. However, when considering sub-threshold reactions, namely wheal diameters of 2 to 3 mm, the T4-T5 mix was reactive in 6 cases at the concentration of 10 μM.

## Discussion

Although the efficacy of SIT has been well established, there are still concerns on its safety and on the particularly long duration of the treatment [4,5]. Despite improved conventional protocols of SIT, severe anaphylactic reactions may sometimes occur even in well-trained centers. The duration of the therapy is another factor of discouragement for patients. The possibility to shorten the treatment period, and to decrease the number of injections, together with improved safety, would provide a considerably more satisfying strategy for allergists to treat patients.

There have been so far several attempts to decrease the allergenicity of immunotherapy products, including so-called allergoid preparations [36-38], and more recently T cell peptides, allergen fragments or mutated allergens. T cell epitopes are short enough to stay unrecognized by IgE in most cases, but are well recognized by T cell receptors. They appear to induce tolerance in volunteers injected intradermally with multi-T cell epitope preparations [16]. They did not induce immediate-type reactions but late respiratory symptoms including dyspnea, which occurred a few hours after the injections [39,40]. Long synthetic peptides overlapping by 5 to 20 amino acids (COPs), as applied in the current study, were initially developed from PLA2, the major bee venom allergen [17,18]. PLA2 hypersensitive volunteers did not react to intradermal tests with PLA2 derived long synthetic peptides. A phase I clinical trial in bee venom hypersensitive patients was safe and induced only transient late allergic reactions (sensation of heat in two volunteers) and was immunogenic, namely inducing a significant rise in PLA2 specific IgG4 [20]. In the present study, the same approach was applied to Bet v 1, the dominant allergen from birch pollen, a very common respiratory allergy in Northern Europe. Three sets of peptides were obtained, two consisting in three overlapping peptides T1-T2-T3 and T6-T7-T8, another in two overlapping peptides T4-T5. Their sequences were derived from the most representative Bet v 1 isoform, rBet v 1.01A. The interest of COPs relies in part in their capacity to cover all potential T cell epitopes known to be spread all along the Bet v 1 molecule [24], as previously observed also with phospholipase A2 [17]. In contrast to other hypoallergenic fragments [15,41], overlapping of COPs prevents the loss of T cell epitopes possibly located at the site of cleavage of the fragments, thus avoiding the risk of any HLA restriction [17]. Furthermore, the purity of the product, compared to pollen extracts, insures the absence of contaminating allergens [42], endotoxins [43] and potentially reduces the risk to induce IgE sensitization to uncontrolled components [44].

Peptide IgE binding capacity was first evaluated in competition ELISA with human sera. The endpoint of the assay was at least a ten-fold lower IgE binding activity of peptides as compared to recombinant Bet v 1, an objective largely fulfilled here. Indeed, in comparison to rBet v 1, we can estimate that AllerT showed at least a 10^7^-fold lower ability to bind IgE. This value is below values previously reported for hypoallergenic Bet v 1 variants, including folded variant [45], fragments [46] or trimers [47].

We examined in parallel the capacity of AllerT to induce anaphylactic reaction in a murine model. BALB/c mice sensitized to rBet v 1 were not affected by the intraperitoneal injection of AllerT (at a five-fold higher concentration than rBet v 1), whereas the i.p. injection of rBet v 1 induced a sharp drop in rectal temperature, a surrogate marker of anaphylaxis. These *in vivo* data largely supported the results obtained with the rat basophil leukemia cell line *in vitro*.

Although we cannot fully insure that anaphylactic reaction was dependent on the induction of IgE, anti-rBet v 1 specific murine IgE reached high titers after the fourth subcutaneous injection as measured by ELISA (Figure 3A). In parallel we also observed a strong increase in rBet v 1 specific IgG1 and IgG2a. Massive IgG1 activation has been shown to induce anaphylactic shock in mice in the absence of IgE [48]. In contrast IgG2a, a possible murine equivalent of IgG4 [49], may antagonize allergen-IgE binding and protect against anaphylaxis. Despite the presence of both IgG1 and IgE against rBet v 1 in sensitized mice, our combination of COPs, AllerT, was unable to induce anaphylaxis in contrast to the recombinant allergen.

The low, if any, IgE binding activity of COPs as compared to rBet v 1 was confirmed by a functional human basophil activation assay as well as in an assay based on huFcϵRI transfected RBL. Using individual peptides as well as their mixes, only background basophil or RBL activation was noted in presence of sera from birch pollen hypersensitive patients. Finally in *in vivo* assays, twenty volunteers were tested by skin prick tests for reactivity to Bet v 1, to birch pollen extract as well as to the five COPs and their mixes (AllerT, T4-T5 mix). As shown in Table 3, a decrease in skin sensitivity of about 10 to 100 folds was observed. This was true for T1 to T5 COPs individually. Interestingly, in 6 patients, the mix of T4-T5 COPs was associated with a skin response of 1 to 3 mm in diameter, in one patient for COP T5 only and in two patients for COP T1. Together with residual IgE binding activity in ELISA competition assays, these results suggest a partial refolding of T4 and T5, which potentially reconstitute the original IgE epitopes in solution. This has already been observed for other proteins [50,51]. This example shows the importance to select for peptides with no detectable IgE binding either in competition ELISA or degranulation tests before going to human trial since possible re-association may occur after *in vivo* injection as exemplified by the skin prick tests reported in this study.

## Conclusions

Taken together, these experiments demonstrate that an allergen specific immunotherapy strategy based on the injection of selected COPs derived from Bet v 1 may be applied to patients with hypersensitivity to birch pollen. The presented data, performed both in murine and human models, indicate that the binding of the selected COPs to IgE, if any, is extremely limited. COPs were a) safe after subcutaneous injection in sensitized mice since they did not induce anaphylaxis, b) unable to activate human basophils and c) did not induce prick test reactions. As compared to birch pollen derived COPs T4 and T5, the mix of COPs T1-T2-T3 (AllerT) appears as an optimal candidate mix for a phase I clinical trial in birch pollen hypersensitive patients with allergic rhinoconjunctivitis.

## Competing interests

Céline Pellaton, Yannick Perrin, Caroline Boudousquié, Nathalie Barbier, Jacqueline Wassenberg, Giampietro Corradin, Anne-Christine Thierry, and Régine Audran have no conflict of interest. Christophe Reymond is employee of Anergis SA, Epalinges, Switzerland. François Spertini and Christophe Reymond are founder members of Anergis SA and possess shares in the society.

## Authors’ contribution

All authors have been involved in drafting the manuscript and revising it critically; all authors have given approval of the submitted version of the paper. Furthermore, FS and CR contributed to acquisition of funding, GC to peptide synthesis, CB, NB, and ACT to establishing key laboratory techniques and revising illustrations.
